# Prevalence and distribution of human papillomavirus (HPV) in Luoyang city of Henan province during 2015–2021 and the genetic variability of HPV16 and 52

**DOI:** 10.1186/s12985-022-01759-5

**Published:** 2022-03-04

**Authors:** Xiuli Wang, Shuizhong Han, Xingwei Li, Xiaochuan Wang, Shan Wang, Li Ma

**Affiliations:** 1 Department of Blood Transfusion and Clinical Laboratory, No.989 Hospital of the Joint Logistic Support Force of Chinese PLA, Luoyang, Henan Province China; 2 College of Veterinary Medicine, Northwest A&F University, Yangling, Shaanxi China

**Keywords:** Human papillomavirus, Genotypes, Sublineage, Variations

## Abstract

**Background:**

Persistent high-risk Human papillomavirus (HPV) subtypes infection has been implicated as a causative of cervical cancer. Distribution and genotypes of HPV infection among females and their variations would assist in the formulation of preventive strategy for cervical cancer. The purpose of the present study is to investigate the prevalence of HPV among females in central China.

**Methods:**

The distribution and genotypes of HPV among 9943 females attending the gynecological examinations in central of China during 2015–2021 were investigated. HPV genotypes were detected using a commercial kit. Nucleotides sequences of L1, E6 and E7 genes in HPV16 or HPV52 positive samples collected in 2021 were amplified by polymerase chain reaction (PCR). Variations of L1, E6 and E7 in HPV16 and HPV52 were gained by sequencing and compared with the reference sequence. Sublineages of HPV16 and HPV52 were determined by the construction of phylogenetic tree based on L1 gene.

**Results:**

The overall prevalence of HPV infection was 22.81%, with the infection rate of high-risk human papillomavirus (HR-HPV) was 19.02% and low-risk human papillomavirus (LR-HPV) was 6.40%. The most top five genotypes of HPV infection were HPV16 (7.49%), HPV52 (3.04%), HPV58 (2.36%), HPV18 (1.65%) and HPV51 (1.61%). Plots of the age-infection rate showed that the single HPV, multiple HPV, HR-HPV, LR-HPV infection revealed the same tendency with two peaks of HPV infection were observed among females aged ≤ 20 year-old and 60–65 year-old. The predominant sublineage of HPV16 was A1 and B2 for HPV52. For HPV16, The most prevalent mutations were T266A (27/27) and N181T (7/27) for L1, D32E for E6 and S63F for E7 in HPV16. For HPV52, all of the nucleotide changes were synonymous mutation in L1 (except L5S) and E7 genes. The K93R mutation was observed in most HPV52 E6 protein.

**Conclusions:**

The present study provides basic information about the distribution, genotypes and variations of HPV among females population in Henan province, which would assist in the formulation of preventive strategies and improvements of diagnostic probe and vaccine for HPV in this region.

## Background

Cervical cancer is the fourth most commonly diagnosed and leading cause of cancer death among females worldwide, with an estimated 570,000 cases and 311,000 deaths in 2018 [1]. Around 85% of women diagnosed and 87% of women who died from cervical cancer live in the developing countries [2]. Human papillomavirus (HPVs) are found in the cervical carcinoma tissues of most patients and the oncogenic HPVs are regarded as the major cause of cervical cancer. In China, it was reported that there are estimated 110,650 new cancer cases and 36,714 cancer deaths are attributable to HPVs infection in 2015, of which cervical cancer accounted for 85.6% and 78.1% [3].

HPVs are small non-enveloped double-stranded DNA viruses that belong to the genus *Alpha-Papillomaviridae* family [4]. The HPVs genomes are about 7.2–8.0 kb and contain eight open reading frames (ORFs), including: the presumptive early (E1–E2, E4–E7), late (L1 and L2) and Long Control Region (LCR) [5–7]. The continued expression of the E6 and E7 genes is related to induce cellular immortalization, transformation, and carcinogenesis [6]. The E6 and E7 proteins would be candidate for the development of therapeutic vaccines [8]. The L1 protein is the primary composition of HPVs and can self-assemble into virus like particles (VLPs) [9]. The first generation commercial HPV vaccines are based on the recombinant expression of L1 protein in system [10, 11]. Human immunized with commercial HPV vaccines can acquire robust immunity against the homology genotype [9]. The polymorphisms of HPV L1 gene affect the generation of neutralization antibody of different binding affinities [12].

More than 200 different HPVs genotypes have been characterized according to the greater than 10% difference within the L1 gene sequence [4, 5, 13]. Based on their association with cervical cancer, HPVs genotypes are classified into high-risk HPV (HR-HPV, including HPV52, 18, 31, 33, 35, 39, 45, 51, 52, 56, 58 and 59) and low-risk HPV (LR-HPV, including HPV6, 11, 40, 42, 43 and 44) [13]. Individual HPV genotypes are referred to as variants or subtypes when less than 10% difference is within the L1 gene sequence [5]. The HPVs variants are grouped into distinct lineages and sublineages based on nucleotides alignments and phylogenetic analyses [5]. Usually, the L1 nucleotides differences among HPV lineages and sublineages are 1.0–10.0% and 0.5–1.0%, respectively [5].

The most common HPV genotypes in invasive cervical cancer were 16, 18, 31, 33, 35, 45, 52 and 58 worldwide [14]. In different regions of China, the most prevalent HPV genotypes are different. For example, in Beijing and Jiangsu province, HPV16 was the predominant type, in Shanghai and Zhejiang province, HPV52 was the most common type [15–18]. HPV16 has been classified into four major lineages and nine sublineages, including: (1) A, includes A1-3 (European [E]) and A4 (Asian [As]) sublineages; (2)B, includes B1 (African-1 [Afr1a]) and B2 (African-1 [Afr1b]); (3) C (African-2 [Afr2a]); and (4) D, includes D1 (North American 1 [NA]), D2 (Asian-American 2 [AA]) and D3 (Asian-American 1 [AA]) [5, 19]. The lineages and sublineages of HPV16 sequences have geography characteristics [20–22]. HPV52 has been classified into four major lineages and seven sublineages, including A (A1 and A2), B (B1 and B2), C (C1 and C2) and D [5]. Sublineages and single nucleotide polymorphism (SNPs) in HPV, especially the E6 and E7, are associated with the disease status of HPV persistent infection and the elevated risk of cervical carcinoma [13, 23–27].

In the present study, the distribution and genotypes of HPV infection among females in Henan province during 2015–2021 were investigated based on commercial HPV test kit. As the L1 gene plays an important role in the classification of HPV sublineage, so L1 gene of HPV16 and HPV52 were sequenced and applied to phylogenetic analysis. The E6 and E7 are the major oncogenes and the variations are correlated with the progression of cervical lesions. The L1, E6 and E7 genes of HPV16 and HPV52 were sequenced and compared with the reference HPVs strain. The distribution and genotypes of HPVs would assist on the formulation of the vaccination program and preventative strategies against cervical cancer. Variations of the HPVs genetic may be useful for the analysis of cervical cancer risk, even provide crucial information for the development of diagnostic tools and vaccine design.

## Methodology

### Study population and samples

From May 2015 to May 2021, consecutive cervical swabs from females who attended in the gynecological outpatient in the 989 Hospital of Joint Service Support Force of Chinese PLA, Military Training Medical Research Institute of the Whole Army, which is located in Luoyang city, Henan province, central of China were collected. The hospital is open for non-military people in China. The female was eligible to be study if she: (a) had no use of vaginal medication or washing in the previous 72 h; (b) had not had sexual intercourse in the previous 24 h; (c) was not presently during menstruation; (d) had no use of acetic or iodine. Before collection, the females were informed and a written consent was received. The study protocol was approved by the institutional ethics committee in the 989 Hospital of Joint Service Support Force of Chinese PLA, Military Training Medical Research Institute of the Whole Army (Grant No.: LLSC20150305).

### HPV genotyping

The DNA of the samples was extracted by kit and then applied to HPV genotypes by flow-through hybridization and gene chip (Chaozhou Hybribio Limited Corporation, Chaozhou, China) according to the manufacturer’s instruction. The PCR reaction volume was 25 μl, which included 1 μl of template DNA, 23.25 μl buffer and 0.75 μl *Taq* DNA polymerase (Chaozhou Hybribio Limited Corporation, Chaozhou, China). The PCR program was as follows: initial denaturation step at 95°C for 9 min; followed by 40 cycles of 95°C for 20 s, 55°C for 30 s, 72°C for 30 s, and a final 72°C extension for 5 min. The genotyping was performed via hybridization of the PCR products to gene chip containing 37 genotype-specific oligonucleotides and the genotype was analyzed using HybriMax (Chaozhou Hybribio Limited Corporation, Chaozhou, China). The chip can identify 37 genotypes, including 19 LR-HPV (namely 6, 11, 34, 40, 42, 43, 44, 54, 55, 57, 61, 67, 69, 70, 71, 72, 81, 83 and 84) and 18 HR-HPV (16, 18, 26, 31, 33, 35, 39, 45, 51, 52, 53, 56, 58, 59, 66, 68, 73 and 82). The final results were detected by colorimetric change on the chip under direct visualization and blue-purple spots were recognized as HPV positive.

### HPV sequencing

Samples collected in 2021 that were only positive for HPV16 or HPV52 were chosen and processed for the variant analysis of L1, E6 and E7 genes by sequencing*.* To amplify the full length of the L1, E6 and E7 genes, primers were designed based on published HPV16 (GeneBank NC 001526) and HPV52 (GeneBank NC 001592) sequences. The primers used for the amplification of L1, E6 and E7 genes were shown in Table1 and synthesized by Sangon Biotech, Inc. (Shanghai, China). The PCR reaction volume was 50 μl, which included 2 μl of template DNA, 25 μl 2 × PrimeSTAR Max Premix(Takara Biotechnology Co., LTD, Dalian, China), 2 μl of each primer and 19 μl of ultrapure water. The PCR program was as follows: initial denaturation step at 94°C for 10 min; followed by 30 cycles of 95°C for 30 s, 55°C for 30 s, 72°C for 30 s, and a final 72°C extension for 10 min. The PCR products were visualized on 1% agarose gels stained with GoldView TM Nucleic Acid Stain. Identified plasmids containing the L1, E6 or E7 genes were used as positive control and the reaction mixture containing no template as negative control. The targets fragments were then purified using TIANgel Midi Purification Kit (TIANGEN BIOTECH, China) and ligated into p-EASY-Blunt cloning vector (TransGen Biotech, China) according to manufacturer’s instruction. The recombinant plasmids were then transformed into Trans1-T1 Phage Resistant Chemically Competent Cells (TransGen Biotech, China) according to manufacturer’s protocols. The positive clones containing the recombinant plasmids were sent to Sangon Biotech, Inc. (Shanghai, China) for sequencing.

**Table 1:** Primers used for the amplification of HPV16 and HPV52 L1, E6 and E7 genes

Primer name	Start codon	Sequence 5–3	Amplicon size (bp)
HPV16 L1 1F	4636	GACCAAGCTCCTTCATTAATTCCT	1051
HPV16 L1 1R	5686	GGCATCAGAGGTAACCATAGAAC	
HPV16 L1 2F	5393	GCTATGGACTTTACTACATTACAGGC	971
HPV16 L1 2R	6363	TTTACAAGCACATACAAGCACATA	
HPV16 E6 F	7119	TTATGCACCAAAAGAGAACTGCA	502
HPV16 E6 R	7620	GGTGTATCTCCATGCATGATTACAGC	
HPV16 E7 F	7595	GCTGTAATCATGCATGGAGATACACCT	316
HPV16 E7 R	4	GCAGGATCAGCCATGGTAGATTAT	
HPV52 L1 1F	5457	CCACTATGTCCATTGAGTCAGGTC	1013
HPV52 L1 1R	6469	CCTGGCACAGGGTCACCTAAG	
HPV52 L1 2F	6205	GTCCTCCCCTACAGCTCATTAA	977
HPV52 L1 2R	7181	CACAGACAATTACCCAACAGAC	
HPV52 E6 F	31	GGGTGTAACCGAAAACGG	545
HPV52 E6 R	575	ATAGTTGCTTTGTCTCCACG	
HPV52 E7 F	354	GGGAAAACATTAGAAGAG	591
HPV52 E7 R	944	TTGTTTTTCTATTATTGCCTCTA	

### Molecular characterization and phylogenetic analysis of HPV16 and HPV52

The variations of the L1, E6 and E7 genes and proteins were gained by the comparison and numbered with the reference strain HPV16 (GeneBank NC 001526) and HPV52 (GeneBank NC 001592) by DNAStar (Madison, WI, USA). Variants between the studied and reference sequence were noted and the frequencies were calculated.

Phylogenetic analyses the L1 gene of HPV16 and HPV52 were constructed using the MEGA (version 6.0). A neighbor-joining algorithm was employed and Kimura 2-parameter distance neighbor-joining trees were built with 1000 bootstrapped replicates. Furthermore, HPV16 and HPV52 reference strains that deposited into NCBI GenBank Database were included to represent each lineage [5]. For HPV16, the reference strains include A1 (K02178), A2 (AF536179), A3 (HQ644236), A4 (AF534061 and LC368960), B1 (AF536180), B2 (HQ644298), C (AF472509), D1 (HQ644257), D2 (AY686579) and D3 (AF402678). For HPV52, the reference strains include A1 (X74481), A2 (HQ537739), B1 (HQ537740), B2 (HQ537743), C1 (HQ537744), C2 (HQ537746) and D (HQ537748).

### Statistical analysis

SPSS version 19.0 (IBM, Armonk, NY, USA) was used to assess the significance of differences detected in the frequency of HPV infections among groups. The χ^2^ test was used to compare the prevalence of HPV infection. A p-value < 0.05 was considered statistically significant.

## Results

### Characteristics of the study participants

**Fig. 1:**
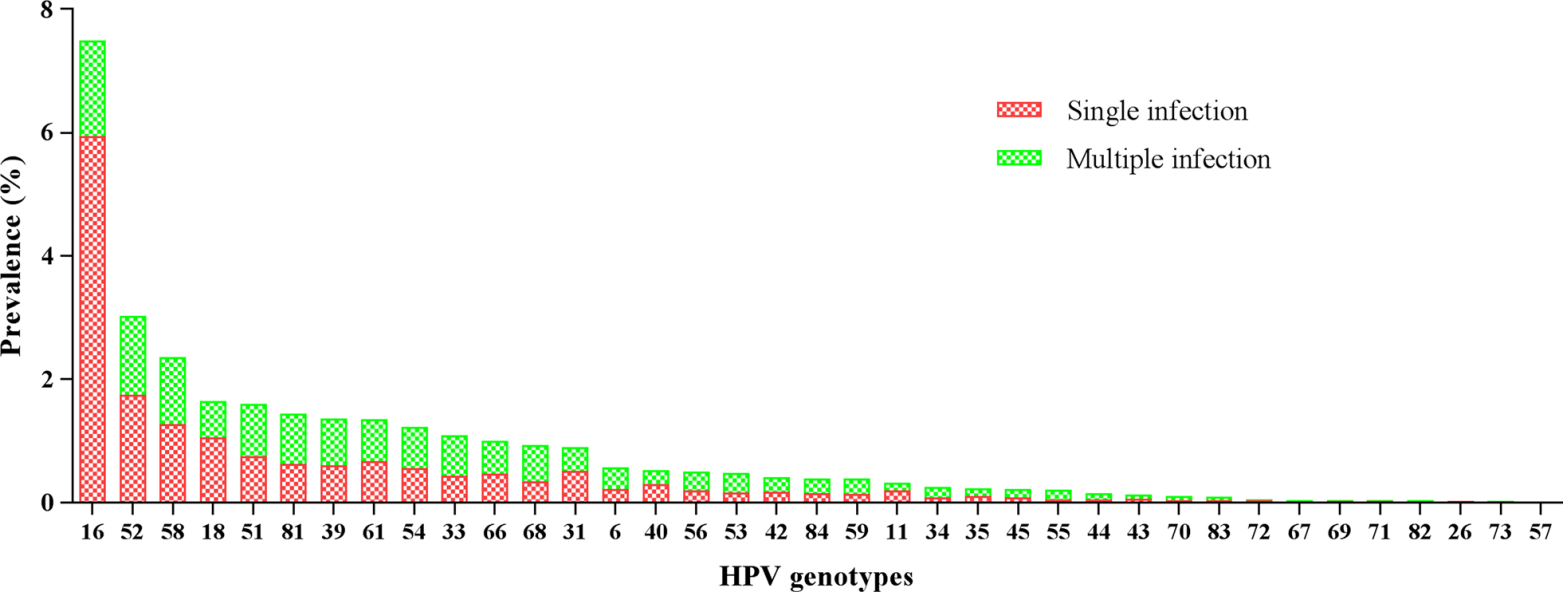
Prevalence of the HPV genotypes in single and multiple infections

A total of 9943 females (ranging from 18 to 90 years old, the median age is 41.15 ± 11.41) underwent outpatient gynecological examinations met the participation criteria. Overall, 22.81% of females (2268/9943) were found to be HPV positive for any HPV DNA, of whom 19.02% (1891/9943) were found to be HR-HPV infection (including samples are HR-HPV only and both positive for HR-HPV and LR-HPV), substantially higher than the LR-HPV infection (including samples are positive for LR-HPV only and both positive for LR-HPV and HR-HPV) (6.40%, 636/9943) (*P* < 0.01) (Table 2). The top five commonly identified HPV genotypes were all HR-HPV, including HPV16 (7.49%, 745/9943), HPV52 (3.04%, 302/9943), HPV58 (2.36%, 235/9943), HPV18 (1.65%, 164/9943) and HPV51 (1.61%, 160/9943). The most prevalent LR-HPV subtypes were as follows: HPV81 (CP8304) (1.46%, 145/9943), HPV61 (1.36%, 135/9943), HPV54 (1.24%, 123/9943), HPV6 (0.58%, 58/9943) and HPV40 (0.53%, 53/9943) (Fig. 1).

**Table 2:** The prevalence of HPV infection in all the specimens (n = 9943)

HPV genotype	Sample numbers	Single infection (%)	Multiple infections (%)	Double infections (%)	Triple infections (%)	Four or more infections (%)
Any HPV type	2268(22.81%)	1731(17.41%)	537(5.40%)	369(3.71%)	104(1.05%)	64(0.64%)
LR-HPV only	377(3.79%)	342(3.44%)	35(0.35%)	30(0.30%)	4(0.04%)	1(0.01%)
HR-HPV only	1632(16.41%)	1389(13.97%)	243(2.44%)	193(1.94%)	34(0.34%)	16(0.16%)
LR-HR HPV	259(2.60%)	0	259(2.60%)	146(1.47%)	66(0.66%)	47(0.47%)

LR-HPV low-risk human papillomavirus, HR-HPV high-risk human papillomavirus, Low risk types, LR-HR HPV low-risk human papillomavirus and high-risk human papillomavirus

Among the 9943 females, 17.41% (1731/9943) were infected with single HPV subtype, whereas 5.40% (537/9943) were infected with multiple HPV subtypes. In the multiple infection groups, the highest infection rate was double infection 3.71% (369/9943) (Table 2). In the single infection group, HPV16 (7.49%) was the most prevalent subtype, followed by HPV52 (3.04%), HPV58 (2.36%) and HPV18 (1.65%). In the multiple HPV infections group, the most prevalent subtypes were as follows: HPV16 (1.55%), HPV52 (1.29%), HPV58 (1.09%), HPV51 (0.84%), HPV81 (0.82%) (Fig. 1).

### Prevalence of HPV infection in different age groups and years

**Fig. 2:**
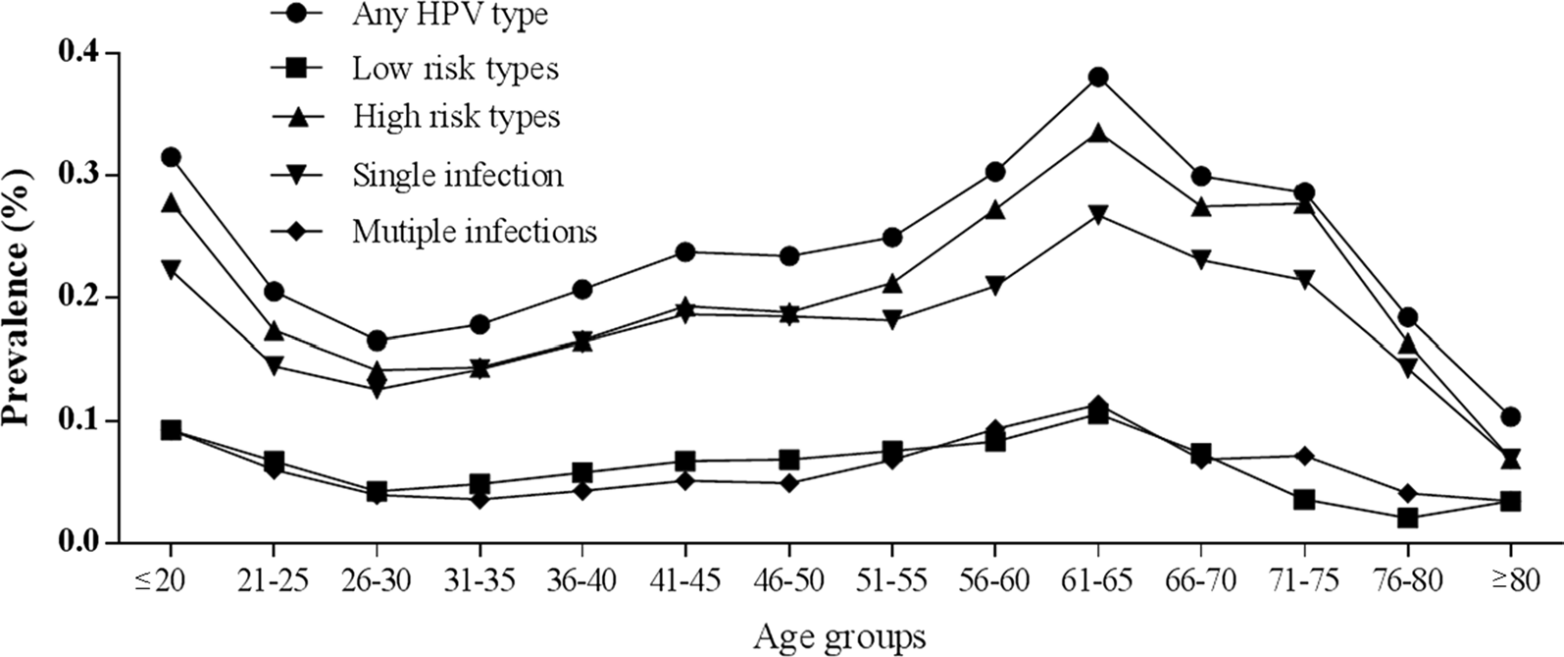
Prevalence of the HPV infection types in different age groups

To evaluate the relationship between HPV infection with the age, females were divided into fourteen age groups. There are significant differences in the HPV infection rates among females in different age groups (χ^2^ = 134.563, *P* < 0.01). Among the 2268 females with HPV infection, there were two peaks of HPV infection, the first was in the ≤ 20 year-old group (31.48%, 17/54) and the second was in the 61–65 year-old group (38.04%, 151/397). All of the HR-HPV, LR-HPV, single infection and multiple infection groups showed the same tendency with the “Any HPV type” infection in different age groups (Fig. 2).

**Fig. 3:**
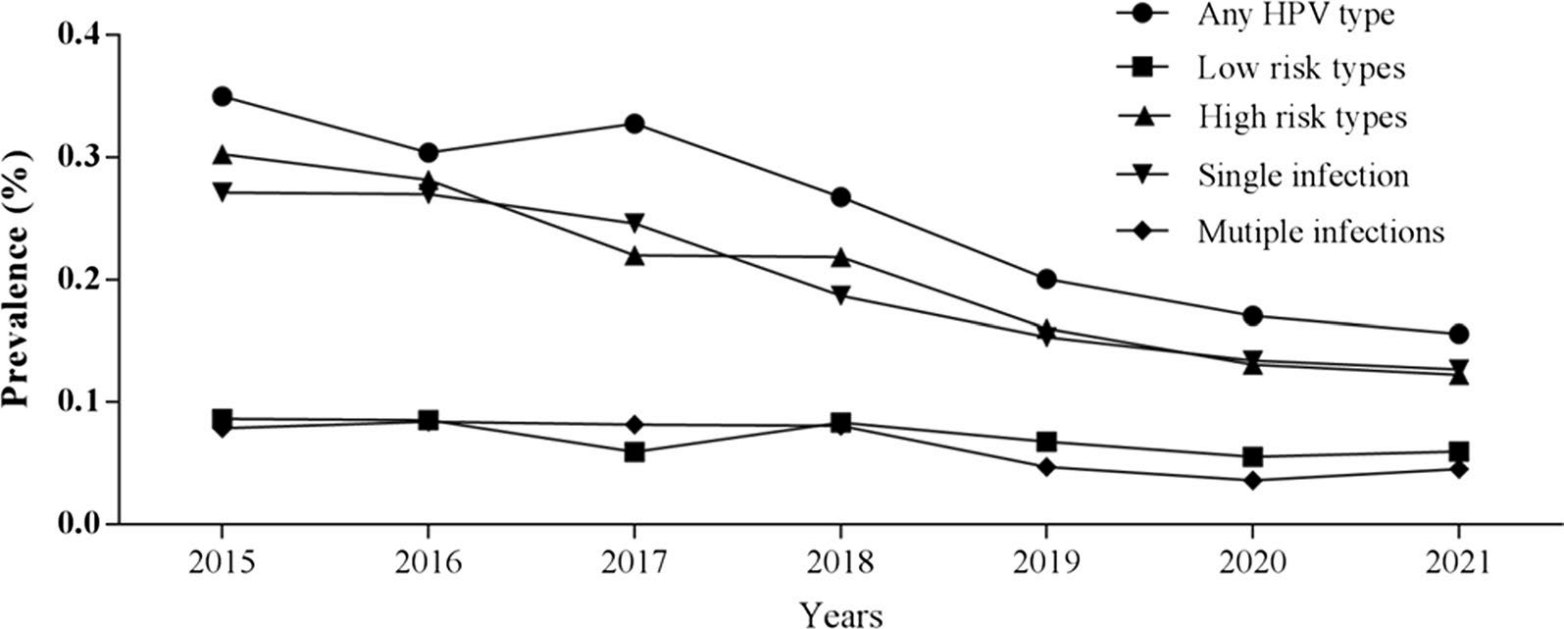
Prevalence of the HPV infection types during 2015 and 2021

For all of the groups, the HPV infection rates are significant differences (*P* < 0.01) during 2015 and 2021. The highest HPV infection rates were observed in 2015, with the any HPV type was 34.98%, the high risk types was 30.26%, the single infection was 27.11% and then declined gradually (Fig. 3).

### HPV16 and HPV52 L1 gene nucleotide variations and amino acid mutational analysis

Twenty-seven HPV16 L1 genes were sequenced successfully and twelve different sequences were submitted to GenBank (MZ546238-MZ546249). The twelve HPV16 L1 gene shared 99.6–99.9% identities with the reference sequence (NC 001526). The variation sites and frequencies of HPV16 L1 gene are shown in Table3. Twelve variations (0.8%, 12/1596) were identified in the 1596 bp L1 gene. Specially, four changes were non-synonymous mutations, including: H76Q (1/27), N181T (7/27), E240D (1/27) and T266A (27/27). The A5570G gene variation was found in all of 27 samples and brought the amino acid change from threonine to alanine (T266A). Another high frequent variation was A5316C (25.9%, 7/27), which changed asparagine into threonine (N181T). The most common synonymous mutations in L1 gene were A5803C (66.7%, 18/27) and G6196A (70.4%, 19/27).

**Table 3:** Nucleotide sequence mutations of HPV16 L1 genes

	Domain: HPV16 L1 sequence													
nt	4	5	5	5	5	5	5	5	5	5	5	6	n	Sublineage
	9	0	3	4	5	5	6	7	8	9	9	1		
	3	0	1	9	2	7	8	9	0	6	7	9		
	3	2	6	4	8	0	3	4	3	2	4	6		
NC_001526	A	T	A	A	A	A	T	A	A	C	G	G		Protype
MZ546238						G	C		C			A	5	A1
MZ546239						G			C				7	A1
MZ546240	G					G	C		C			A	1	A1
MZ546241					C	G							1	A1
MZ546242						G	C		C			A	1	A1
MZ546243		G	C			G	C		C			A	1	A1
MZ546244						G				T		A	1	A3
MZ546245			C			G				T		A	1	A3
MZ546246						G		T	C			A	2	A1
MZ546247						G		T	C		A	A	1	A1
MZ546248				C		G						A	1	A4
MZ546249			C			G						A	5	A4
reference aa	K	H	N	E	R	T	D	T	S	S	E	L		
aa position			1	2	2	2	3	3	3	3	4	4		
	5	7	8	4	5	6	0	4	4	9	0	7		
	3	6	1	0	2	6	3	0	3	6	0	4		
aa mutations		Q	T	D		A								
Frequency	1	1	7	1	1	27	8	3	18	2	1	19	27	

Positions without variants are marked with a dash, whereas positions with variants are indicated by a letter

Fifteen HPV52 L1 genes were sequenced successfully and five different nucleotide sequences were gained by comparison and then submitted to GenBank (OL589507-OL589511). The five sequences shared 99.0%-99.9% identities with the reference sequence (NC 005192). Compared with the NC 001592, nineteen nucleotide changes were identified among the fifteen sequences (Table 4). All of the changes in HPV52 L1 gene were synonymous mutation except L5S (6.7%, 1/15). Seven synonymous mutations in HPV52 L1 gene, including G6110A, T6701G, T6764C, A6794G and C6824T were found in 93.3% (14/15) samples. The G6218A was detected in all of fifteen sequences.

**Table 4:** Nucleotide sequence mutations of HPV52 L1 genes

	Domain: HPV52 L1 sequence																				
nt	5	5	5	5	5	5	6	6	6	6	6	6	6	6	6	6	6	7	7	n	Sublineage
	5	7	7	8	9	9	0	1	2	4	6	7	7	7	8	9	9	0	1		
	7	7	7	2	0	7	8	1	1	4	9	0	6	9	2	1	3	5	1		
	8	1	7	8	9	2	3	0	8	3	8	1	4	4	4	7	2	2	2		
NC_001592	T	A	A	A	A	T	G	G	G	C	G	T	T	A	C	C	A	G	G		Protype
OL589507						C		A	A			G	C	G	T	A		A		1	B2
OL589508		G				C		A	A			G	C	G	T	A		A		11	B2
OL589509	C				G		A		A	T	A					A	C		A	1	C2
OL589510		G	G			C		A	A			G	C	G	T	A		A		1	B2
OL589511		G		G		C		A	A			G	C	G	T	A		A		1	B2
reference aa	L	T	V	K	P	I	R	Q	Q	A	E	V	F	K	Y	V	I	R	S		
					1	1	1	1	2	2	3	3	4	4	4	4	4	4	5		
		6	7	8	1	3	7	8	1	9	7	7	0	1	2	5	5	9	1		
aa position	5	9	1	8	5	6	3	2	8	3	8	9	0	0	0	1	6	6	6		
aa mutations	S																				
Frequency	1	13	1	1	1	14	1	14	15	1	1	14	14	14	14	15	1	14	1	15	

Positions without variants are marked with a dash, whereas positions with variants are indicated by a letter

### HPV16 and HPV52 E6-E7 gene nucleotide variations and amino acid mutational analysis

Eighteen HPV16 E6 and E7 genes were sequenced successfully. Thirteen different E6 sequences (MZ546266-MZ546278) and E7 sequences (MZ546295-MZ546307) were obtained and the identity was 99.4–100% for E6 gene and 98.7–100% for E7 gene compared with the HPV16 reference sequence (NC 001526). Compared with the reference sequence, nine nucleotide mutations were observed in the HPV16 E6 genes and eight were non-synonymous mutations (Table 5). The most frequently non-synonymous mutation in HPV E6 genes were T7220G (A) (5/18), which made D32E mutation. Eight nucleotide changes occurred in the HPV16 E7 genes with four were non-synonymous mutations. The most frequently observed non-synonymous in HPV16 E7 genes were C7791T (S63F) (Table 5).

**Table 5:** Nucleotide sequence mutations of HPV16 E6-E7 genes

	Domain: HPV16 E6 sequence											Domain: HPV16 E7 sequence								
nt	7	7	7	7	7	7	7	7	7	n	nt	7	7	7	7	7	7	7	7	n
	1	1	2	2	2	2	2	3	5			6	6	7	7	8	8	8	8	
	3	3	1	1	2	3	6	9	4			8	8	0	9	0	3	8	8	
	6	7	0	8	0	0	1	2	9			8	9	8	1	2	2	5	8	
NC001526	G	A	C	G	T	G	G	T	G		NC001526	A	A	G	C	T	C	T	T	
MZ546266					G					1	MZ546295		G		T			C	C	1
MZ546267	A								A	2	MZ546296			A	T					2
MZ546268					G					2	MZ546297		G		T				C	2
MZ546269										2	MZ546298						T			2
MZ546270								G		1	MZ546299				T					1
MZ546271										2	MZ546300			A	T					2
MZ546272										1	MZ546301				T	C				1
MZ546273	A				A					1	MZ546302			A						1
MZ546274	A	G			A					1	MZ546303			A						1
MZ546275				A						1	MZ546304					C				1
MZ546276			G				A	G		1	MZ546305	C								1
MZ546277				T						1	MZ546306					C				1
MZ546278						C				2	MZ546307									2
Reference aa	K	R	T	D	D	E	R	L	R			N	N	E	S	L	R	C	S	
aa position									1											
			2	3	3	3	4	9	4			2	2	3	6	6	7	9	9	
	4	5	9	2	2	6	6	0	2			9	9	5	3	7	7	4	5	
aa mutations		G	S	N/Y	E	Q	Q	V	Q			H	S		F		C			
Frequency	4	1	1	2	5	2	1	2	2	18		1	3	6	9	3	2	1	3	18

Positions without variants are marked with a dash, whereas positions with variants are indicated by a letter

Fifteen HPV52 E6 and E7 genes were sequenced and thirteen sequences were identical (Table 6). The most prevalent non-synonymous mutations in E6 genes was A379G (14/15) and cause the amino acid to change from Lysine to arginine (K93R). For E7 sequences, the high frequent mutations was C751T and A801G, both were synonymous mutations.

**Table 6:** Nucleotide sequence mutations of HPV52 E6-E7 genes

nt	Domain: HPV52 E6 sequence			n		Domain: HPV52 E7 sequence										n
	3	3	5			5	6	7	7	7	7	7	7	8	8	
	5	7	3			7	6	0	0	2	3	4	5	0	4	
	0	9	0			3	2	6	7	7	3	2	1	1	8	
NC_001592	G	A	A		NC_001592	T	C	A	G	T	C	G	C	A	T	
OL589512	T	G		14	OL589514								T	G		14
OL589513	T		G	1	OL589515	A	T	G	A	G	T	A		G	G	1
Reference aa		K	R			T	T	S	S	Y	H	D	L	Q	R	
aa position			1													
	8	9	4				3	5	5	5	6	6	6	8	9	
	3	3	3			7	7	2	2	9	1	4	7	3	9	
aa mutations		R					I	D	D	D	Y	N				
Frequency	15	14	1			1	1	1	1	1	1	1	14	14	1	15

Numbers of the variation in the domain of the L1 genes were statistics in bracket

### Phylogenetic analysis

**Fig. 4:**
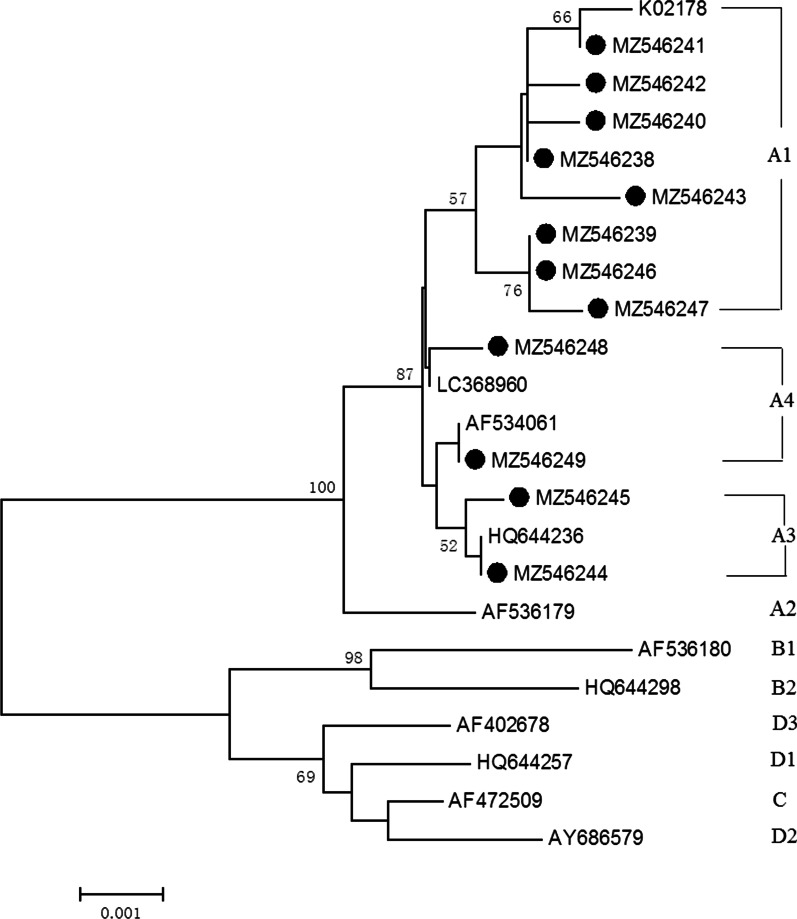
Neighbor joining phylogenetic tree generated using nucleotide sequences of the HPV16 L1 gene**.** Legend: Study sequences are labeled in dots, others without dots are reference strain, including: A1 (K02178), A2 (AF536179), A3 (HQ644236), A4 (AF534061 and LC368960), B1 (AF536180), B2 (HQ644298), C (AF472509), D1 (HQ644257), D2 (AY686579), D3 (AF402678). Phylogenetic trees were constructed by the Neighbor-Joining method and the Kimura 2-parameter model by MEGA 6.0 package. Only bootstrap values above 50% are displayed in the branches

**Fig. 5:**
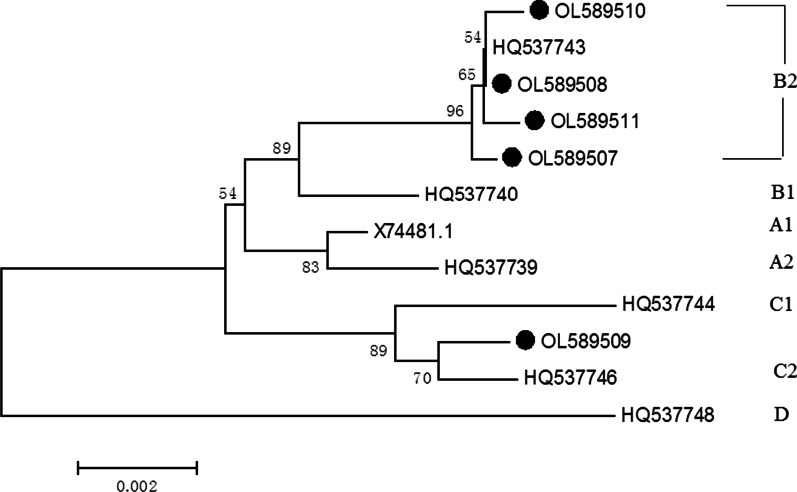
Neighbor joining phylogenetic tree generated using nucleotide sequences of the HPV52 L1 gene. Legend: Study sequences are labeled in dots, others without dots are reference strain, including: A1 (X74481.1), A2 (HQ537739), B1 (HQ537740), B2 (HQ537743), C1 (HQ537744), C2 (HQ537746), D (HQ537748). Phylogenetic trees were constructed by the Neighbor-Joining method and the Kimura 2-parameter model by MEGA 6.0 package. Only bootstrap values above 50% are displayed in the branches

Phylogenetic analysis based on the full length of HPV16 and HPV52 L1 genes are shown (Figs. 4 and 5). All of the twenty-seven HPV16 L1 sequences, 70.4% (19/27) were A1 sublineage, 22.2% (6/27) were A4 sublineage and 7.4% (2/27) were A3 (Fig. 4). All of the fifteen HPV52 L1 sequences, 93.3% (14/15) were B2 sublineages (Fig. 5).

## Discussion

Globally, cervical cancer is the fourth most common malignancy in females around world and contributes 530 000 new cases per year [1]. Persist infection with HR-HPV has been identified as a major risk factor for cervical cancer. The prevalence of high-risk HPVs infections, such as HPV16, HPV18, HPV52, increased with severity of cervical lesions [28]. In the present study, a retrospective survey of HPV infection among 9943 females who underwent gynecological outpatient clinic during 2015 to 2021 was conducted in a located hospital. Though it is a military hospital, it is open for non-military people. Occupation of females in this study was unclear, which may take deviations in statistical analysis. In this study, the overall prevalence of HPV in Henan province was 22.81%, which was similar to Beijing (21.06%), Zhejiang (22.8%), Sichuan (24.01%) and Jiangxi (22.49%) provinces [29–32]. However, significant differences on the HPV infection rates were observed in other provinces, such as Shaanxi (30.21%), Xinjiang (9.34%) and Yunnan (7.6%) provinces [33–35]. Many factors, including sex, age, ethnicity and socioeconomic status, may contribute to the significant difference on the HPV infection rates in different areas [36].

In the present study, HPV16 was the most prevalent HR-HPV genotype (7.49%), followed by HPV52 (3.04%), HPV58 (2.36%), HPV18 (1.65%) and HPV51 (1.61%). In some regions of China, for example Beijing, the top prevalent HR-HPV genotypes in patients were as follows: HPV16 (27.34%), HPV58 (12.52%), HPV52 (11.89%) and HPV51 (6.33%) [16]. In Shanghai, HPV52 was the most predominant genotype (3.58%), followed by HPV16 (2.85%), HPV58 (2.64%), HPV53 (1.81%) and HPV39 (1.46%) [17]. The top five LR-HPV in the present study were HPV81 (1.46%), HPV61 (1.36%), HPV54 (1.24%), HPV6 (0.58%) and HPV40 (0.53%). Commercial 2-valent (HPV16 and HPV18), 4-valent (HPV 6, 11, 16 and 18) and 9-valent (HPV 6, 11, 16, 18, 31, 33, 45, 52 and 58) HPV vaccine have been approved by the National Medical Products Administration since 2016. However, the proportion of females who were willing to receive HPV vaccine are relative low [37]. The present study may provide valuable data to inform cervical cancer screen and the implementation of HPV vaccination in Henan province. For the development of HPV multiplicity vaccine, vaccines contained HPV51 and HPV81 would be more efficiency for the prevention of HPV infection in China.

The relationship between population age and HPV infection rate was investigated. Two peaks of infection rate were observed in the any type HPV, HR-HPV, LR-HPV, single and multiple HPV infection among ≤ 20 and 61–65 year-old females, which have been observed in other reports [15, 29, 38]. The first peak of HPV infection occurred in the ≤ 20 years group (31.5%, 17/54), with 9.3% (5/54) was LR-HPV and 27.8% (15/54) was HR-HPV infection. The high HPV infection rate was obvious in females aged ≤ 20 years was partly due to the limited sample numbers. On the other hand, high sexual activity and lack of immunity to HPV may contribute to the high HPV infection rate [39]. The second peak was observed in the 61–65 year-old groups (38.0%, 151/397), which consisted of 10.6% (42/397) LR-HPV infection and 33.5% (133/397) HR-HPV infection. It was assumed that viral persistence or reactivation of latent HPV due to the physiologic and immunologic deregulation caused by hormone fluctuations may explain the high HPV infection rate around menopausal women [40]. The present study would assist in the formulation of preventive strategy for cervical cancer and more inspections, including cytology and even colposcopy, should be proceed among women aged > 60 years for the prevention of cervical cancer.

The L1 protein is the major capsid protein and able to induce immune response [12]. Phylogenetic distance and amino variations of the L1 protein have an effect on the immune efficiency of HPV vaccines [11, 41]. The uncontrolled expression of E6 and E7 proteins inactivates the p53 and pRb tumor suppressor proteins and is associated with the HPV persist infection [42]. HPV variants and nucleotide mutations have been suggested to affect the oncogenic potential of HPV persistent infection [22–24, 43]. Thus, the L1, E6 and E7 sequences of the most predominant HPV (HPV16 and HPV52) were selected to study lineage phylogeny and the genetic polymorphisms.

Based on the L1 genes, the predominant HPV16 sublineage in Henan province was A1 in 2021. In other areas in China, such as Beijing city, Zhejiang and Yunnan province, the sublineage A4 was the most common genotype [44–47]. It was reported the sublineage A4 were associated with more severity disease status than A1-3 sublineage in Chinese females and higher risk of cancer [22, 23, 48, 49]. Compared with the reference (NC001526), four non-synonymous mutations were found in HPV16 L1 protein, including H76Q (1/27), N181T (7/27), E240D (1/27) and T266A (27/27). The amino mutations N181T (7/27) and T266A (27/27) were also found in other provinces, such as Shanghai and Sichuan province [47, 50, 51]. Synonymous mutations in L1 gene, including G6196A (19/27, 70.4%), A5803C (18/27, 66.7%) and T5683C (8/27, 29.6%), had also been reported in Sichuan province [51]. In the present study, the most prevalent HPV52 sublineage in Henan province was B2. It was reported that the HPV52 sublineage B2 predominated in Asian, while in Africa, Americas and Europe, lineage A was the most common lineage [52]. Compared with HPV52 lineage A, the B2 sublineage showed a higher risk [52].

For HPV16, the most frequent non-synonymous mutations found in E6 gene was D32E (7/18). Although the D32E mutation in E6 protein did not change the B-cell epitopes, the gene variation altered the other gene profiles [53, 54]. It was suggested that the D32E amino mutation had a significant correlation with the persistent HPV16 infection in females [47]. The S63F was the most prevalent non-synonymous mutations in E7 genes. It was reported that the S63F mutation was more frequent in women with carcinoma cancer [44]. The reason was assumed that the S63F variation had an influence on the E7 epitopes and caused viral persistence and cervical cancer [44]. Compared with the HPV52 reference sequence (NC 001,592), the K93R was the only one non-synonymous mutation. The K93R mutation was also observed in other HPV52 isolates in China [55, 56]. Though the K93R mutation did not increase the cell immortalization ability of HPV52, a higher colony formation and greater cell migration ability was observed when compared to HPV52 prototype [57]. The synonymous mutation C751T and A801G were observed in other report and the roles need be further studied [56].

## Conclusion

In summary, the present study provides basic information about the distribution, genotypes and variations of HPV among females population in central China, which would assist in the formulation of preventive strategies and improvements of diagnostic probe and vaccine for HPV in this region.

## Data Availability

All data and materials described in manuscript are available.

## Abbreviations

HPV: Human papillomavirus
LR-HPV: Low-risk human papillomavirus
HR-HPV: High-risk human papillomavirus
